# Tunable Electromagnetic and Microwave Absorption Properties of Magnetic FeNi_3_ Alloys

**DOI:** 10.3390/nano13050930

**Published:** 2023-03-03

**Authors:** Yu Zheng, Mei Wu, Congyi Qian, Yuxin Jin, Wei Xiao, Xiaohui Liang

**Affiliations:** Hangzhou Dianzi University, Hangzhou 310018, China

**Keywords:** FeNi_3_ alloy, microwave absorption, reflection loss, effective absorption bandwidth

## Abstract

Magnetic materials have a very broad application prospect in the field of microwave absorption, among which soft magnetic materials become the focus of magnetic materials research because of their high saturation magnetization and low coercivity. FeNi_3_ alloy has been widely used in soft magnetic materials because of its excellent ferromagnetism and electrical conductivity. In this work, FeNi_3_ alloy was prepared by the liquid reduction method. The effect of the filling ratio of FeNi_3_ alloy on the electromagnetic properties of absorbing materials was studied. It is found that the impedance matching ability of FeNi_3_ alloy is better when the filling ratio is 70 wt% than that of other samples with different filling ratios (30–60 wt%), showing better microwave absorption characteristics. When the matching thickness is 2.35 mm, the minimum reflection loss (RL) of FeNi_3_ alloy with a 70 wt% filling ratio reaches −40.33 dB, and the effective absorption bandwidth is 5.5 GHz. When the matching thickness is between 2 and 3 mm, the effective absorption bandwidth ranges from 7.21 GHz to 17.81 GHz, almost covering the whole X and Ku bands (8–18 GHz). The results show that FeNi_3_ alloy has adjustable electromagnetic properties and microwave absorption properties with different filling ratios, which is conducive to selecting excellent microwave absorption materials.

## 1. Introduction

With the rapid development of information technology in social life, the influence of electromagnetic waves on society has grown deeply. Electromagnetic interference is gradually becoming a serious problem that affects our daily life [1,2,3,4,5]. Using microwave-absorbing materials is a good solution to solving this problem, which could absorb electromagnetic waves [6,7]. The microwave-absorbing material not only requires high absorption strength but also needs to have a thinner thickness and lighter weight so that it can be used effectively in practical applications [8,9]. Microwave-absorbing materials have many uses in different fields. For example, in the military, they can be used as a paint for fighter jets to absorb microwaves emitted by radars, achieving effective concealment [10,11].

According to the different absorption mechanisms, microwave-absorbing materials can be divided into two types: dielectric loss and magnetic loss materials [12]. Magnetic loss materials include ferrite, carbonyl iron and other magnetic materials [13,14]. Ferrite (such as Fe_3_O_4_ and NiFe_2_O_4_) is a material often used in the field of microwave absorption. The absorbers made of ferrite-absorbing material have a thin thickness, but the synthesis process of ferrite is difficult, and the effective bandwidth of the absorber is not enough to cover various frequency bands [15,16]. However, Fe-based alloy composites can perfectly improve these performance deficiencies. Yang et al. used a chemical method to prepare the FeCo nanosheet. The composite material comprising epoxy resin can obtain a reflection loss value of −43 dB at 8.1 GHz and a thickness of 1.8 mm [17]. Liu et al. used an electroless plating method to prepare FeNi alloy-coated flake graphite. When the thickness of the composite material was 1.2 mm, it obtained a minimum absorption RL of −43.7 dB at 12.5 GHz [18]. Deng et al. synthesized the Mxene/HFO (hollow Fe_3_O_4_) mixture via electrostatic assembly. With a thickness of 1.56 mm, the composite has a minimum absorption RL of −63.7 dB [5].

In recent years, many studies have researched iron-based alloys, such as iron–nickel and iron–cobalt alloys, as well as further research on coatings with iron-based alloys [19,20,21,22,23]. Among them, the soft magnetic nickel alloy has become popular in the research of modern microwave-absorbing materials because of its higher saturation magnetization and lower coercivity [20,24,25]. The different preparation methods of iron–nickel alloys and the changing of the ratio of iron–nickel among them will cause great differences in microwave absorption performance. The synthesis method includes sol–gel and hydrothermal synthesis, etc. In addition, there are many options for microwave-absorbing mixed materials, such as paraffin wax, epoxy resin, etc. Paraffin wax has the advantage of easily passing through waves. For example, the flake FeNi_3_ particles, prepared by Shi et al., used the electrodeposition method with a mixed 30 wt% paraffin, which can obtain a minimum reflection loss of −50.42 dB at 3.44 GHz, and at the same time, when the thickness is 1.3–2.5 mm, it has a particularly wide absorption bandwidth of 4.4–17.52 GHz [26]. Yao et al. used a two-step method with a mixed 70 wt% paraffin to prepare FeNi powder. When the composite material has a thickness of 3 mm, the minimum absorption peak reaches −52.58 dB [27]. Yan et al. used the low-temperature reduction method with a 16.7 vol% paraffin to prepare FeNi_3_ submicron spheres. The absorber thickness was 2.9 mm, and the minimum absorption peak was −61.3 dB at 8.7 GHz [28]. Therefore, iron–nickel is better for microwave absorption.

The research work on the absorption properties of the FeNi alloy composites described above is mostly focused on the material’s properties, and there are few studies on the filling ratio of the composites. However, the filling ratio has a non-negligible influence on the stealth performance of the absorbing material. Therefore, this paper mainly studies the influence of the filling ratio on the absorbing performance of FeNi_3_ alloy by adjusting different filling ratios. The final FeNi_3_ alloy obtained has a good absorbing performance when the filling ratio is 70 wt%. When the matching thickness is 2.35 mm, the minimum reflection loss reaches −40.33 dB, and the effective absorption bandwidth is 5.5 GHz.

## 2. Experimental Procedure

### 2.1. Preparation of FeNi_3_ Alloy Particles

**Scheme 1:**
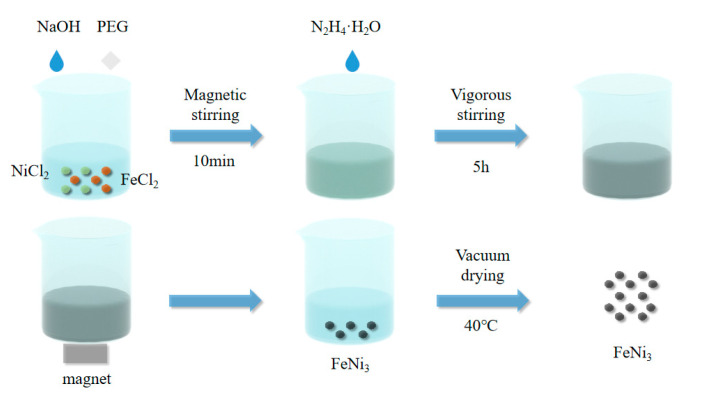
The reaction process diagram of preparing FeNi_3_ alloys.

All the materials were analytically pure (>99.7%) and from Shanghai Maclean Biochemical Technology Co., Ltd. (Shanghai, China). As shown in Scheme 1, the initial materials were 2.5 mmol iron dichloride FeCl_2_·4H_2_O and 7.5 mmol nickel chloride NiCl_2_·6H_2_O. These were dissolved in 80 mL of deionized water H_2_O to form a mixed solution. A total of 12 mL of sodium hydroxide NaOH solution and 1 g of Polyethylene glycol (PEG) were added and magnetically stirred for 10 min, then vigorously stirred with an electric mixer while adding 8 mL of hydrazine hydrates. The solution was continuously stirred for 5 h. After stirring, the obtained solution was washed three times with distilled water and absolute ethanol and finally placed in a vacuum drying oven at 40 °C and vacuum dried to obtain a FeNi_3_ alloy powder sample. Take a certain quality of FeNi_3_ powder and evenly mix it with paraffin wax to make 120 mg of a paraffin wax-FeNi_3_ mixture with FeNi_3_ mass ratios of 30–70 wt%. Among the materials, hydrazine hydrate was used as an inorganic hydride-reducing agent to reduce metal ions from salt solutions or metal organics in solvents. PEG was mainly used as a catalyst to facilitate reactions between ions.

### 2.2. Characterization and Measurements

The X-ray diffractometer (XRD) (Bruker D8 ADVANCE, Germany) was used to analyze the structure information of the alloy powder samples. In this case, the instrument’s detection source for the XRD measurements was Cu target Kα rays, and the range of measurement was from 10° to 90°, with a scanning speed of 2° per minute. A scanning electron microscope (SEM) (Hitachi S4800, USA) and transmission electron microscope (TEM) (JEOL JEM 2100, USA) were used to analyze the morphology, particle size and dispersion of the alloy powder samples. A vibrating sample magnetometer (VSM,) (the Lakeshore 7400, China) was used to measure the magnetic strength of the sample. Energy-dispersive X-ray spectroscopy (EDX) (JEOL JEM 2100, USA) was used for the micro-component analysis. At the same time, the void, atomic proportion and density of the sample were measured. The composites of the alloy powder and paraffin wax with a mass ratio of paraffin wax of 30–70 wt% were pressed into a ring sample with an outer diameter of 7 mm and an inner diameter of 3.04 mm, which was used to measure the electromagnetic parameters with a vector network analyzer (Agilent PNA N5244A, USA).

## 3. Results and Discussion

**Figure 1:**
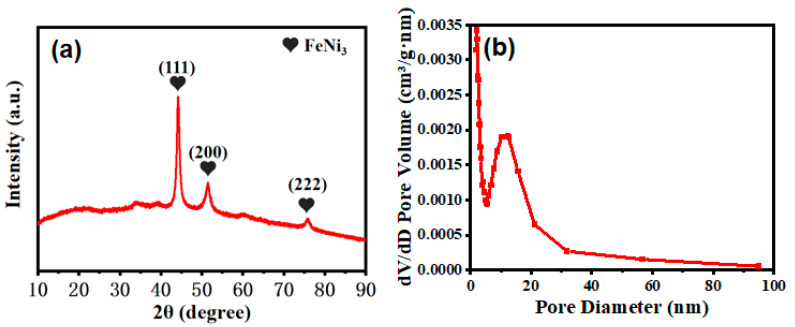
XRD diffraction (**a**) and porosity test results (**b**) of FeNi_3_ alloy sample.

The XRD diffraction of the FeNi_3_ alloy sample is shown in Figure 1a. From the XRD results, there are three diffraction peaks in the range of 10° to 80°, which appear at 44.18°, 51.56° and 75.86°. These can be determined as the (111), (200) and (222) crystal planes of the FeNi_3_ (JCPDS Card No. 38–0419). The crystal system of the FeNi_3_ alloy is a face-centered cubic (FCC) lattice structure, and the lattice parameters are $\dot{a\;}\;$= 3.54, $\dot{b\;}$= 3.54 and $\dot{c}\;$= 3.54. The samples obtained by testing are face-centered cubic (FCC) lattice structures. The crystallite size of the FeNi_3_ alloy was calculated by Debye–Scherer’s formula [29]:

(1) $$D_{m}=\left(K\lambda \right)/\left[\delta \left(2\theta \right)\cos\theta \right]\;$$

where λ represents the wavelength of X-rays, $\delta \left(2\theta \right)$ represents the full width at half maximum of the diffraction peak in the XRD pattern, θ represents the Bragg scattering angle. K is the Scheele constant, and Dm is the grain thickness perpendicular to the grain plane. The Schieler constant is usually calculated between 0.98 and 1.3, but for crystals with cube, sphere, tetrahedral, and octahedral shapes, the calculated value of 0.89 is sufficient to achieve a good fit. Since the samples prepared in this paper are spherical particles, the Schieler constant is set at 0.89. The three strong diffraction peaks in the XRD results are calculated, and the grain size of the FeNi_3_ alloy submicron spheres is about 96 nm [29].

The porosity test result of the FeNi_3_ alloy sample is shown in Figure 1b. As can be seen, most of the voids are distributed in the range of 10–20 nm. At the same time, the density of the FeNi_3_ alloy was prepared by the density tester (G-DenPyc 2900 true, China) with the gas displacement method at 25 °C. The results of the three measurement times show that the density of the prepared sample was 5.84 (g/mL).

**Figure 2:**
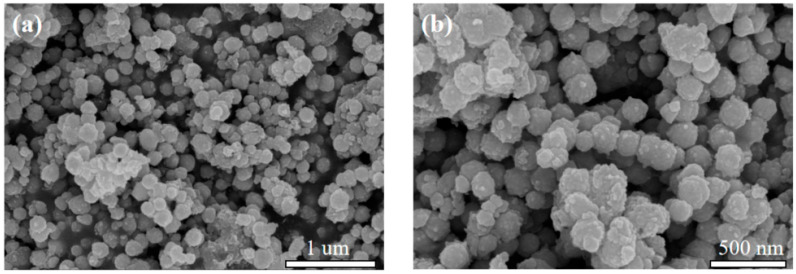
(**a**) Low magnification and (**b**) high magnification SEM images of FeNi_3_ alloy.

The SEM images of the FeNi_3_ composites are shown in Figure 2a,b. By looking at the SEM images, it can be seen that most of the particles appear spherical with well-defined particles. The size of the particle spheres is relatively uniform, with most of them having diameters between 90–100 nm, which corresponds to the XRD results. The surfaces of the granular spheres have rough surfaces with polyhedral edges and corners, and a small number of particles exhibit overlapping lamellar structures, which may be caused by the overlap between particles.

**Figure 3:**
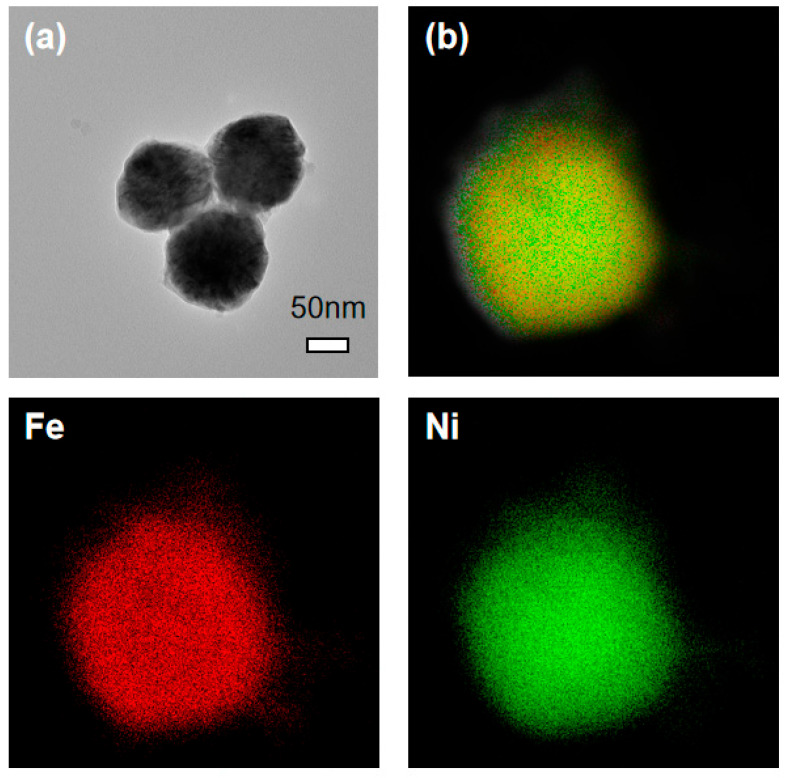
TEM image (**a**) and elemental mapping image (**b**) of FeNi_3_ alloy.

**Figure 4:**
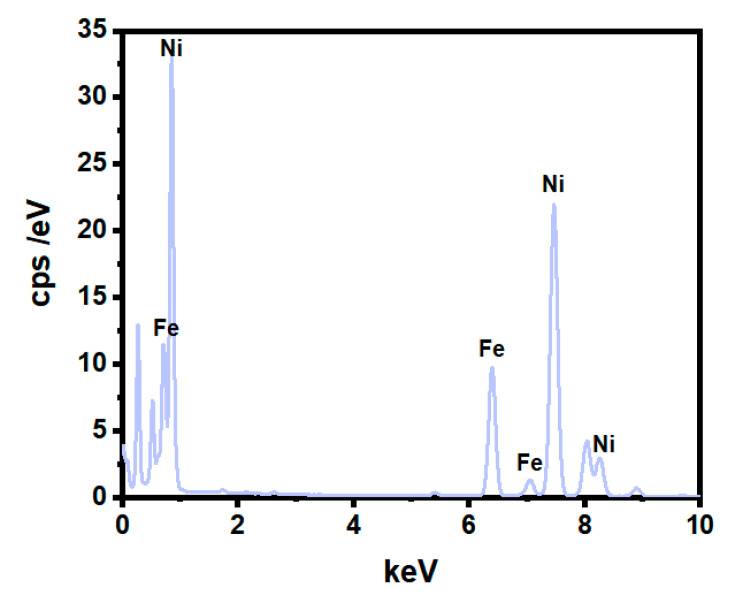
EDX image of FeNi_3_ alloy.

The TEM image of the FeNi_3_ alloy is shown in Figure 3a. The TEM results showed that the prepared FeNi_3_ samples were also spherical nanoparticles with a size of about 100 nm, which was consistent with the SEM results. The elemental mapping and EDX spectral images of FeNi_3_ alloy are shown in Figure 3b and Figure 4, respectively. It can be seen from the EDX that the Fe and Ni elements in the prepared FeNi_3_ sample are evenly distributed, and the content of Ni is more than that of Fe. Meanwhile, the ICP results show that the mass fraction of Fe and Ni in the sample is 23.15% and 71.03%, respectively, with a ratio of nearly 1:3, which proves that the sample prepared by us is indeed FeNi_3_ alloy.

**Figure 5:**
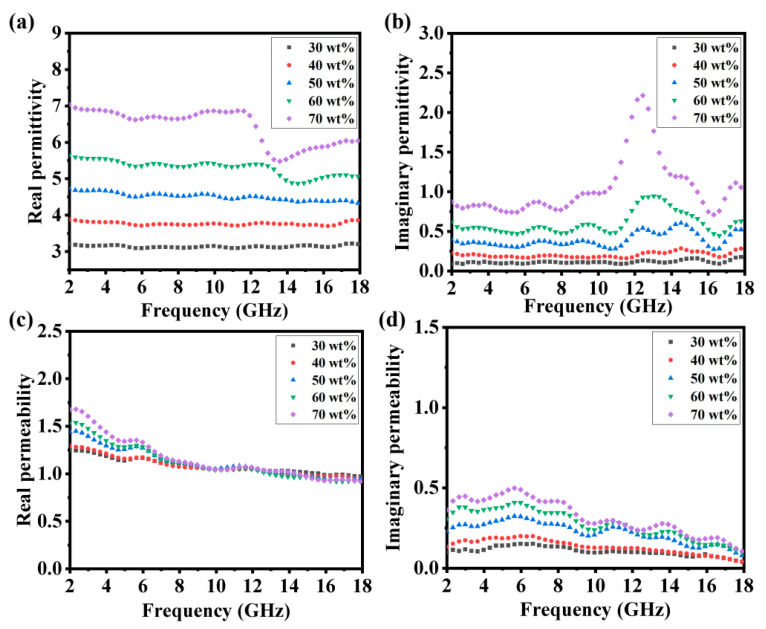
(**a**) The real and (**b**) imaginary parts of the complex permittivity; (**c**) the real and (**d**) imaginary parts of the complex permeability of FeNi_3_ samples with different filling ratios.

The relative complex permittivity and complex permeability of FeNi_3_ alloy with filling ratios of 30–70 wt% were measured in the range of 2–18 GHz (Figure 5a,b). It can be seen that the values of the real and imaginary parts of the relative complex permittivity increase as the filling ratios increase from 30 wt% to 70 wt%. It is known that *ε”* usually represents the storage capacity of electromagnetic wave energy, and *ε″* represents the loss capacity of electromagnetic wave energy [11]. The results show that the FeNi_3_ alloy with a filling ratio of 70 wt% has higher *ε′* and *ε″* than other ratios; thus, the FeNi_3_ alloy with a filling ratio of 70 wt% has better electromagnetic energy storage and loss capacity. It can be explained by the free electron theory [30]. As the mass ratio increases, the *ε″* of the FeNi_3_ alloy composites increases due to the better electrical conductivity of FeNi_3_ alloys. When the filling ratio increases, the composites have higher electrical conductivity, so the *ε″* improves as the filling ratio of FeNi_3_ alloy increases [26]. The dielectric loss in the FeNi_3_ alloy sample resulted from the imaginary part of the permittivity, which is also inseparable from the interfacial polarization phenomenon brought by spherical morphology [28].

**Figure 6:**
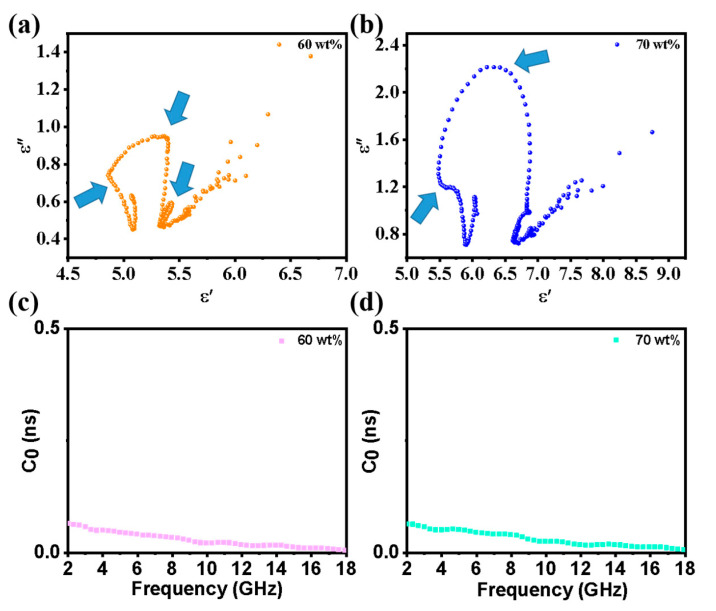
Cole–Cole curve of (**a**) 60 wt%, (**b**) 70 wt% FeNi_3_ alloy mass ratio sample, C_0_ curves of (**c**) 60 wt%, (**d**) 70 wt% FeNi_3_ alloy filling ratios sample.

Figure 6a,b and Figure S1a–c represent the Cole–Cole curves for FeNi_3_ alloys with different filling ratios. It can be seen that there are semicircular curves, and these semicircles indicate the presence of interfacial polarization in the FeNi_3_ alloy samples. Figure 5c,d show the curves of the real and imaginary parts of the FeNi_3_ alloy mixed with paraffin wax with different filling ratios. It can be seen that the real part of the magnetic permeability of all the samples shows a decreasing trend with increasing frequency. The decrease in the complex permeability is due to the dispersion effect; the real part of the permeability will be reduced with the increase of frequency, and the peaks near 5–6 GHz are caused by the natural resonance of the material itself. Meanwhile, the imaginary part of the magnetic permeability in Figure 5d shows a smaller resonance peak, which is favorable for microwave absorption.

**Figure 7:**
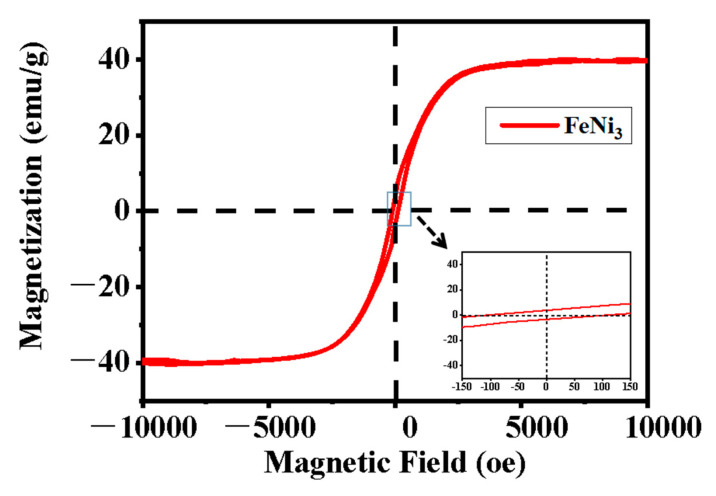
Hysteresis line diagram of FeNi_3_ alloy.

The main factors that cause magnetic loss are eddy current, natural resonance and exchange resonance [31]. In the low-frequency range, the magnetic loss thus results from natural resonance. In Figure 4c, it can be demonstrated how the natural resonance occurred at 6 GHz nearby. In addition, the eddy current loss exists at a lower frequency. The $C_{0}$ curves can be used to judge whether the main factor of the dominant magnetic loss is eddy current loss and the $C_{0}$ curve has been shown in the following calculation formula:

(2) $$C_{0}=\mu^{\prime \prime }{\left(\mu^{\prime }\right)}^{-2}f^{-1}$$

when the C_0_ curve is in the range of 2–18 GHz, if the eddy current losses inside the material dominate the magnetic losses, the curve should behave as a constant without fluctuations and not change with frequency. Figure 6c,d show the C_0_ curves for the FeNi_3_ alloy powders with fillings of 60 wt% and 70 wt%. The values of the C_0_ curve change somewhat with frequency range; therefore, it can be demonstrated that the magnetic losses in the composite are not dominated by eddy current losses but mainly resonance losses. The hysteresis lines for the FeNi_3_ alloy are shown in Figure 7. The hysteresis lines show that the saturation magnetization strength of the FeNi_3_ alloy is 40.13 emu/g, and the coercivity is 111 Oe. It also provides a basis for the magnetic loss capability of the FeNi_3_ alloy material itself.

The reflection loss values of the FeNi_3_ samples are obtained by calculation and simulation of the electromagnetic parameters, and the reflection loss clearly reflects the strength of a material’s microwave absorption capacity. According to transmission line theory, the values of reflection loss can be calculated from the following formulas [32]:

(3) $$Z_{\mathrm{in}}=Z_{0}{\left(\mu_{r}/\varepsilon_{r}\right)}^{1/2}\tan h\left[j\left(2\pi \mathrm{fd}/c\right){\left(\mu_{r}\varepsilon_{r}\right)}^{1/2}\right]$$

(4) $$\mathrm{RL}=20\log|\left(Z_{\mathrm{in}}-Z_{0}\right)/\left(Z_{\mathrm{in}}+Z_{0}\right)|$$

where $Z_{0}$ is the free space impedance, $Z_{\mathrm{in}}$ is the input impedance, f is the frequency of the microwave, c is the speed of light in free space, d is the matching thickness of the absorber, $\varepsilon_{r}$ is the complex dielectric constant, and $\mu_{r}$ is the complex permeability.

**Figure 8:**
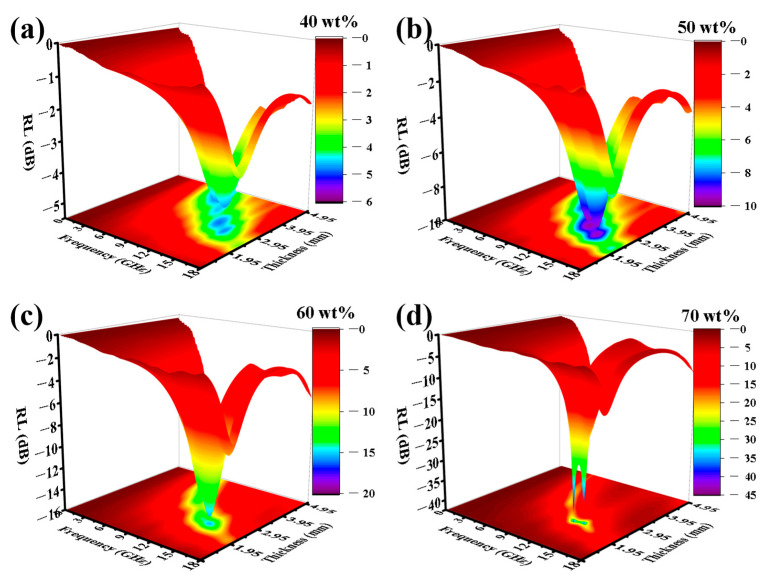
The RL vs. frequency diagram with (**a**) 40 wt%, (**b**) 50 wt%, (**c**) 60 wt%, and (**d**) 70 wt% FeNi_3_ alloy filling ratios.

Figure 8 and Figure S2 show the correlation between the 3D plots of frequency and reflection loss to match the thickness for the ring samples with different FeNi_3_ alloy filling ratios. It can be seen that the microwave absorption performance of the FeNi_3_ sample increases with the gradual increase of the filling ratio of the FeNi_3_ alloy. It can be concluded that when the filling ratio of FeNi_3_ alloy is 30 wt%, the reflection loss of the sample does not reach −10 dB in the full waveband. When the filling ratio of FeNi_3_ alloy is 40 wt%, the minimum reflection loss absorption of −5.45 dB can be obtained when the frequency is at 7.22 GHz and the matched thickness of the sample reaches 5 mm. When the FeNi_3_ alloy fills with 50 wt%, a minimum reflection loss absorption peak of −9.70 dB is obtained when the frequency is 12.16 GHz with a thickness of 2.9 mm. The minimum reflection loss absorption peak of −15.35 dB is obtained when the FeNi_3_ alloy filling ratio is 60 wt%, the frequency is 13.60 GHz, and the matching thickness is 2.5 mm. The FeNi_3_ alloy sample with a 70 wt% filling ratio exhibits the best microwave absorption capability. When the frequency reaches 11.88 GHz, the minimum value of the absorbing material is −40.33 dB, with a strong reflection loss intensity when the matched thickness of the sample is 2.35 mm.

**Figure 9:**
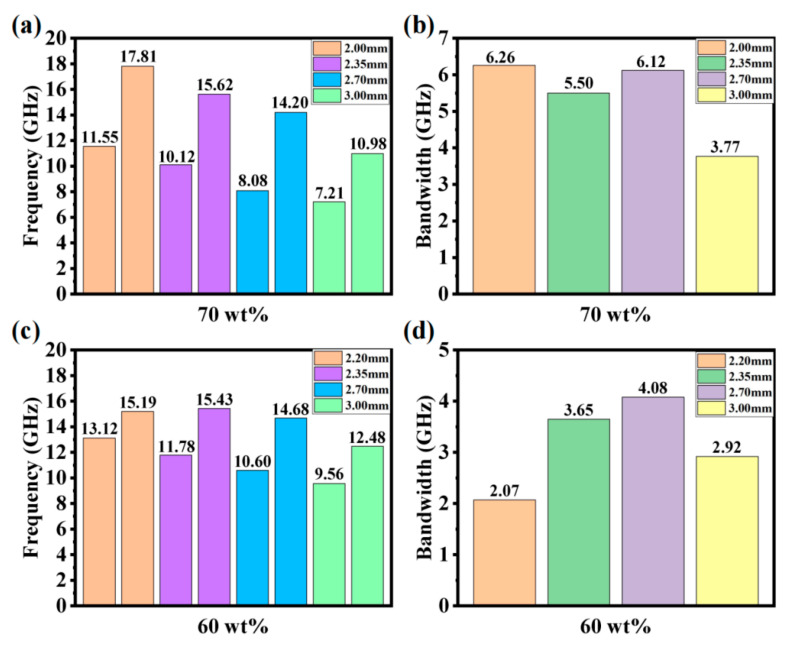
(**a**) Frequency absorption range and (**b**) effective absorption bandwidth of FeNi_3_ sample with 70 wt% filling ratio; (**c**) frequency absorption range and (**d**) effective absorption bandwidth of FeNi_3_ sample with 60 wt% filling ratio.

Figure 9a,c show the frequency range (RL < −10 dB) of the FeNi_3_ samples with 60 wt% and 70 wt% filling ratios when the matching thickness is 2–3 mm. Figure 9b,d show the effective absorption bandwidth for the FeNi_3_ samples with filling ratios of 60 wt% and 70 wt%, respectively. It can be seen that the maximum absorption bandwidth of the FeNi_3_ alloy with a 70 wt% filling ratio can reach 6.26 GHz when the matching thickness is varied, while the sample with a 60 wt% filling ratio can reach, at most, 4.08 GHz. When the matching thickness of a sample with a 70 wt% filling ratio is between 2 and 3 mm (Figure 8a), the frequency range of RL < −10 dB is 7.21 to 17.81 GHz. It has a very wide frequency range, covering almost the entire X and Ku bands (8–18 GHz). The frequency range of RL < −10 dB for the 60wt% filled sample is only 9.56 to 15.43 GHz. Therefore, the FeNi_3_ alloy sample with a 70 wt% filling ratio has a larger absorption bandwidth than the other samples.

**Figure 10:**
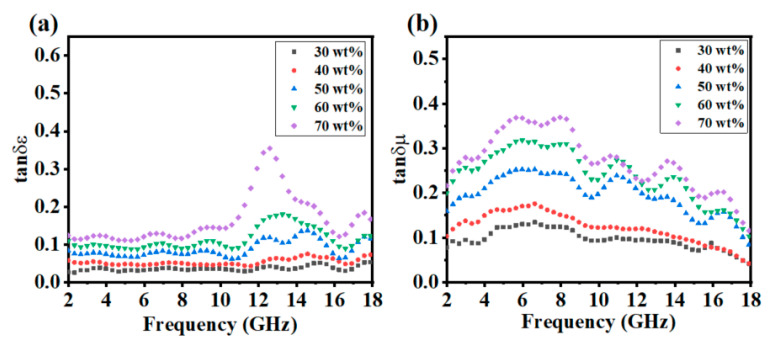
(**a**) The tan δ_ε_ and (**b**) tan δ_μ_ of FeNi_3_ alloy with different filling ratios.

The main factor affecting the absorption performance is the electromagnetic energy loss capacity of the FeNi_3_ alloy. Figure 10 shows the dielectric loss tangent (tan δ_ε_ = ε″/ε′) and magnetic loss tangent (tan δ_μ_ = μ″/μ′) for the FeNi_3_ samples with different filling ratios. It can be seen that the dielectric loss tangent of the sample increases as the filling ratio increases in Figure 10a. When the filling ratio is greater than or equal to 50 wt%, the dielectric loss tangent first remains constant as the frequency increases, and then a resonance peak appears after 10 GHz, where the polarization phenomenon occurs to increase the dielectric loss capability. Figure 10b shows that the magnetic loss tangent of the samples with different filling ratios show a fluctuating decreasing trend with increasing frequency, and resonance peaks also appear at individual locations. As the filling ratios of FeNi_3_ alloy increase, the magnetic loss tangent also increases. Therefore, the synergistic effect of dielectric loss and magnetic loss makes the FeNi_3_ alloy with a filling ratio of 70 wt% have better microwave absorption performance.

**Figure 11:**
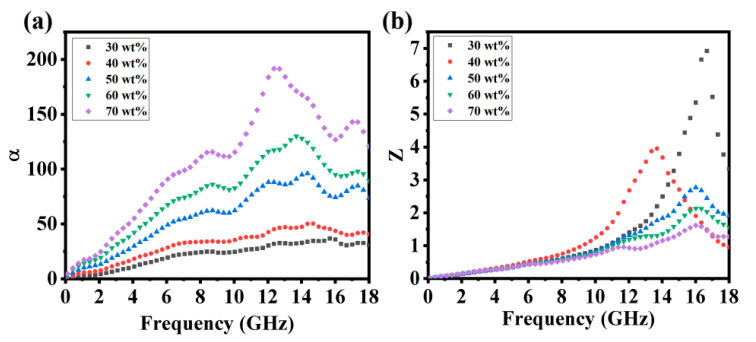
(**a**) Attenuation constant and (**b**) relative input impedance of FeNi_3_ alloy sample with 30–70 wt% filling ratios.

In addition, the other two key factors that determine the microwave absorption capacity are the electromagnetic attenuation ability of the absorber itself and the impedance matching ability [33]. The attenuation constant α determines the attenuation characteristics of the materials, which is presented in the following calculation [34]:

(5) $$\alpha =\frac{\sqrt{2}\pi f}{c}\times \sqrt{\left(\mu^{\prime \prime }\varepsilon^{\prime \prime }-\mu^{\prime }\varepsilon^{\prime }\right)+\sqrt{{\left(\mu^{\prime \prime }\varepsilon^{\prime \prime }-\mu^{\prime }\varepsilon^{\prime }\right)}^{2}+{\left(\mu^{\prime }\varepsilon^{\prime \prime }+\mu^{\prime \prime }\varepsilon^{\prime }\right)}^{2}}}$$

where c represents the speed of light, and f represents the frequency. Figure 11a shows the curves of the attenuation constant α of the FeNi_3_ samples with different proportions. When the FeNi_3_ filling ratio increases, the attenuation constant α also increases. At the same time, they all show an increasing trend with the increase in frequency, indicating the attenuation ability of electromagnetic waves is gradually enhanced. Moreover, the relative input impedance *Z* of the FeNi_3_ sample has been exhibited in the following calculation [35]:

(6) $$Z=\left|Z_{\mathrm{in}}/Z_{0}\right|$$

Figure 11b shows the relative input impedance *Z* of the FeNi_3_ samples with different mass ratios, which gradually decreases with the increase of FeNi_3_ alloy filling ratios. It can be seen that the relative input impedance of the FeNi_3_ alloy with a mass ratio of 70 wt% is closer to 1, which implies a better impedance matching condition. Therefore, the FeNi_3_ alloy with a filling ratio of 70 wt% has better microwave absorption properties.

**Figure 12:**
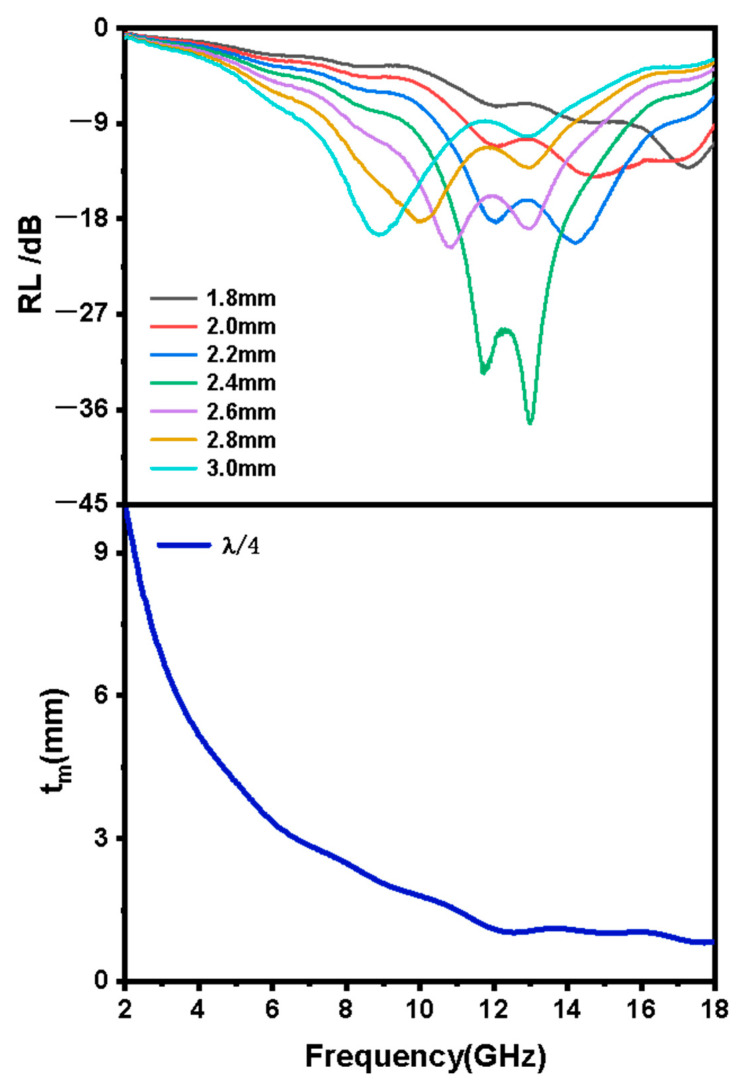
Comparison of various absorbent thicknesses (t_m_) at the frequency for FeNi_3_ alloys with 70 wt% mass ratio sample in λ/4 conditions of maximum RL values (f_m_).

Generally, for absorbing materials, if the thickness of the test sample is an odd time of one-quarter of the wavelength of the incident electromagnetic wave, when the phase angle of the incident electromagnetic wave and reflected electromagnetic wave differs by 180°, part of the energy of the incident electromagnetic wave will be lost, which the quarter-wavelength model can explain. This model has also proven to be suitable for the case where plane waves normally incident on an absorber on a perfect conductor substrate. The thickness that corresponds to the peak frequency of the sample can satisfy the following formula [36,37,38]:

(7) $$t_{m}=\mathrm{nc}/(4f_{m}{\left(\varepsilon_{r}\mu_{r}\right)}^{1/2})\;(n=1,3,5\ldots )$$

where c stands for the velocity of light in free space. In order to determine the maximum RL value that appears at a thickness of 2.35 mm, we performed a simulation of t_m_ under λ/4 occasions for the FeNi_3_ alloy (Figure 12). The quarter-wavelength rule is a vital dissipation element in the thickness design of the absorbent. Blue dots stand for the experimental matching thickness at f_m_, and the blue curve is the simulation thickness using the quarter-wavelength rules. The results show that when the experimental matching thickness is 1.0 mm rather than 2.35 mm, the reflection loss ability is strong and is inconsistent with the simulated thickness. Therefore, the polarization peaks come from the material itself rather than the quarter wavelength. The same phenomenon exists for samples with other filling ratios.

**Figure 13:**
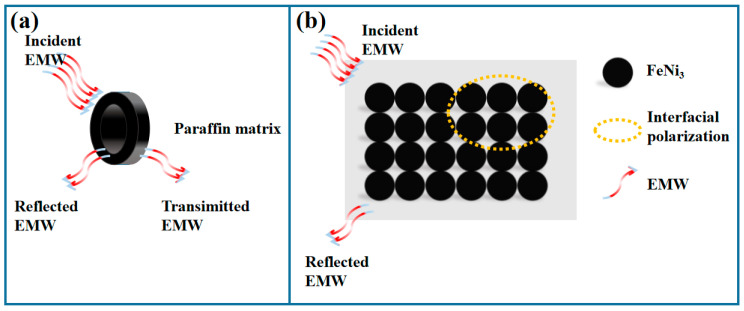
The mechanism of the electromagnetic wave absorption performance of (**a**) the testing ample ring, (**b**) the FeNi_3_ alloy sample.

Figure 13 shows the mechanism of the electromagnetic wave absorption properties of the FeNi_3_ alloy sample. When the electromagnetic wave is incoming, the microwave absorption ring is a whole, in which there are many nanospheres made up. When the wave is incident on the ring, the interaction of many nanospheres causes the wave conduction loss as a whole. When electromagnetic waves propagate between the alloy nanospheres, the accumulation of nanospheres increases the degree of interfacial polarization, which enhances electromagnetic wave absorption. In addition, there is a magnetic loss and dielectric loss to improve microwave absorption.

## 4. Conclusions

In summary, the FeNi_3_ alloy was prepared by the liquid phase reduction method in this study. By controlling the filling ratio of FeNi_3_ alloy in the composites, the influence of the filling ratio on its microwave absorption performance was studied. The results show that the prepared FeNi_3_ alloys have a spherical morphology, and the particle size is uniform. The permittivity and permeability of the FeNi_3_ alloys increased dramatically with the enhanced filling ratios. The dielectric loss and magnetic loss of the FeNi_3_ composite are higher. In addition, the impedance matching is also adjusted with the increase of the filling ratios. Thus, the filling ratio has a certain regulation effect on the microwave absorption performance of FeNi_3_ alloy. Finally, the FeNi_3_ alloy composites showed the best microwave absorption characteristics when the filling ratio was 70 wt%, the absorption performance RL was −40.33 dB at 11.8 GHz, and the effective absorption bandwidth was 5.5 GHz. When the matching thickness was 2~3 mm, the effective absorption bandwidth was 10.2 GHz with a frequency range of 7.2~17.8 GHz. Therefore, the electromagnetic properties of the FeNi_3_ alloy can be adjusted by adjusting the filling ratios, obtaining better impedance matching. This is a candidate way to adjust the absorption performance.

## Data Availability

Data supporting this study’s findings are available from the corresponding authors upon reasonable request.

## Supplementary Materials

The following supporting information can be downloaded at: https://www.mdpi.com/article/10.3390/nano13050930/s1, Figure S1: Cole–Cole curves of FeNi_3_ alloys with 40 wt% and 30 wt% mass ratios, Figure S2: RL values with a 30 wt% mass ratio of FeNi_3_ alloy. Figure S3: Comparison of various absorbent thicknesses (t_m_) at the frequency for FeNi3 alloys with 60 wt% mass ratios sample in λ/4 conditions of maximum RL values (f_m_); Figure S4: Comparison of various absorbent thicknesses (t_m_) at the frequency for FeNi3 alloys with 50 wt% mass ratios sample in λ/4 conditions of maximum RL values (f_m_); Figure S5: Comparison of various absorbent thicknesses (tm) at the frequency for FeNi3 alloys with 40 wt% mass ratios sample in λ/4 conditions of maximum RL values (fm); Figure S6: Comparison of various absorbent thicknesses (t_m_) at the frequency for FeNi3 alloys with 30 wt% mass ratios sample in λ/4 conditions of maximum RL values (f_m_).
