# Supplementary material for: Tunable Electromagnetic and Microwave Absorption Properties of Magnetic FeNi3 Alloys
Source: Nanomaterials (Basel). 2023 Mar 3;13(5):930. doi: 10.3390/nano13050930 (PMC10004822; doi:10.3390/nano13050930)
Supplement: Supplementary file 1 [file nanomaterials-13-00930-s001.zip › nanomaterials-2183214-supplementary.pdf]

nanomaterials

Supplementary Materials

Tunable Electromagnetic and Microwave Absorption Properties of Magnetic $FeNi_3$ Alloys

Yu Zheng, Mei Wu, Congyi Qian, Yuxin Jin, Wei Xiao and Xiaohui Liang *

Hangzhou Dianzi University, Hangzhou 310018, China
* Correspondence: xhliang@hdu.edu.cn

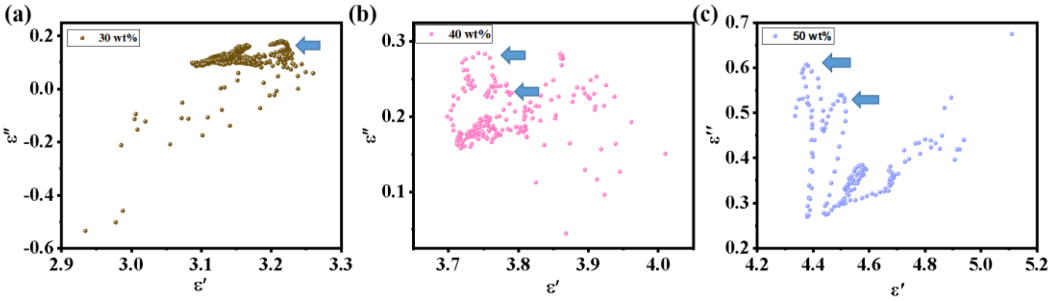

Figure S1. Cole-Cole curves of $FeNi_3$ alloys with 30-50 wt% filling ratios.

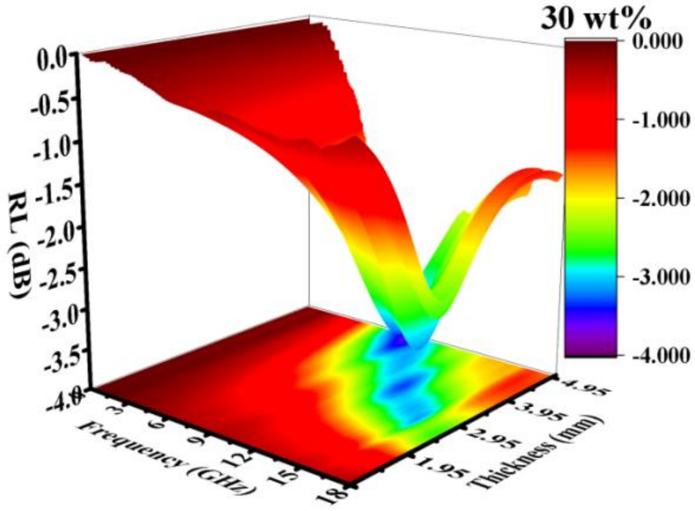

Figure S2. RL values with a 30 wt% filling ratio of $FeNi_3$ alloy.

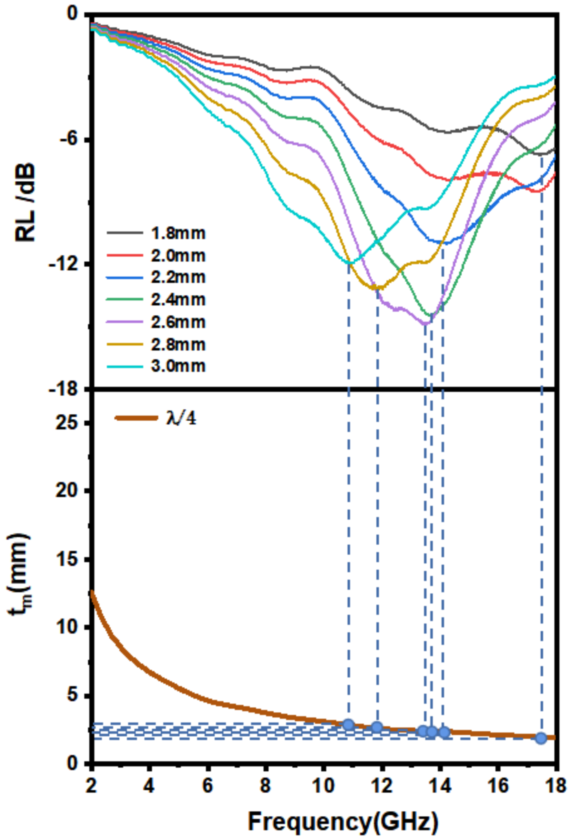

Figure S3. Comparison of various absorbent thicknesses (t_m) at the frequency for $FeNi_3$ alloys with 60 wt% mass ratios sample in $\lambda/4$ conditions of maximum RL values (f_m).

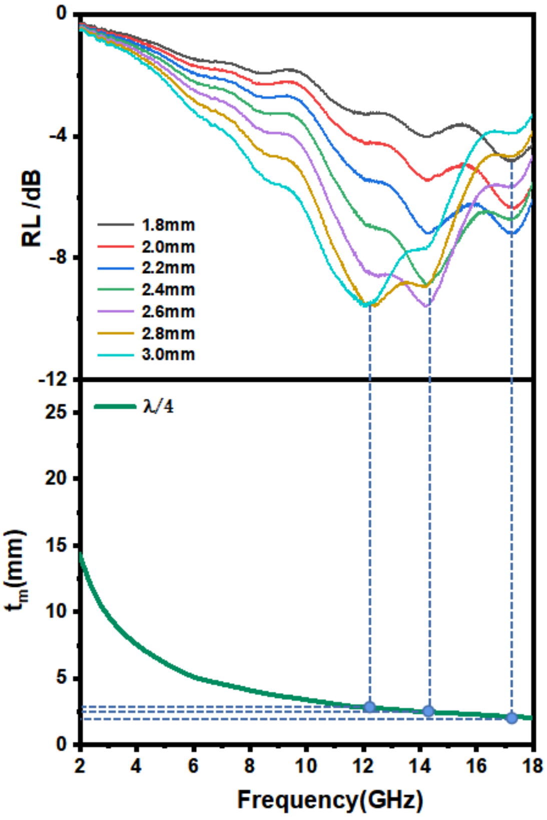

Figure S4. Comparison of various absorbent thicknesses (t_m) at the frequency for $FeNi_3$ alloys with 50 wt% mass ratios sample in λ/4 conditions of maximum RL values (f_m).

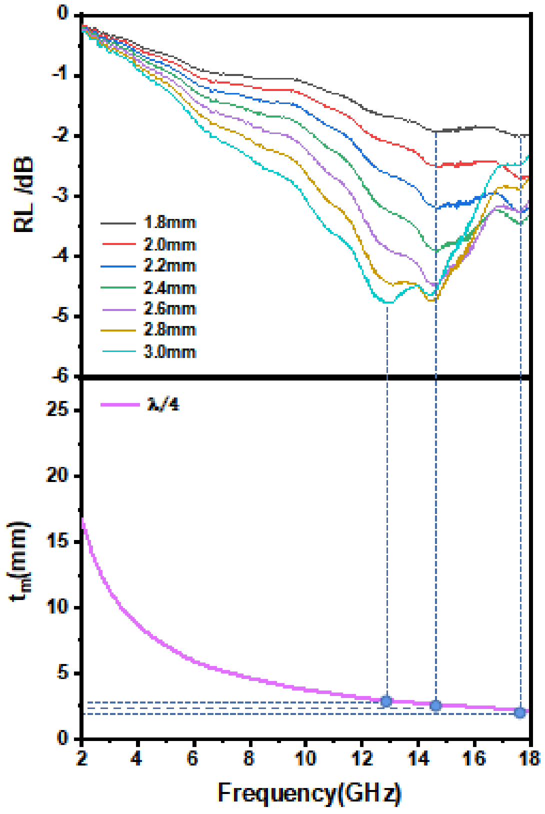

Figure S5. Comparison of various absorbent thicknesses (t_m) at the frequency for $FeNi_3$ alloys with 40 wt% mass ratios sample in λ/4 conditions of maximum RL values (f_m)..

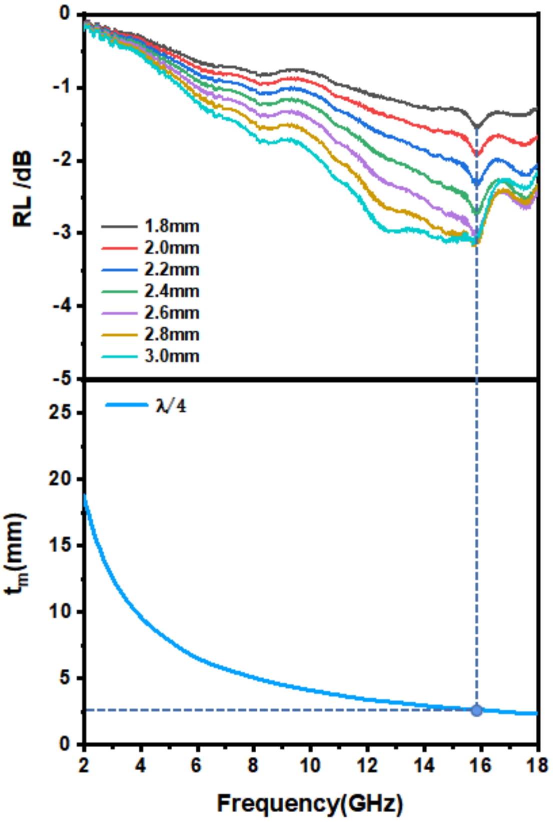

Figure S6. Comparison of various absorbent thicknesses (t_m) at the frequency for $FeNi_3$ alloys with 30 wt% mass ratios sample in $\lambda/4$ conditions of maximum RL values (f_m).
